# 3-Pyridin-2-yl-1*H*-1,2,4-triazol-5-amine

**DOI:** 10.1107/S1600536808042177

**Published:** 2008-12-17

**Authors:** Anton V. Dolzhenko, Geok Kheng Tan, Lip Lin Koh, Anna V. Dolzhenko, Wai Keung Chui

**Affiliations:** a Department of Pharmacy, Faculty of Science, National University of Singapore, 18 Science Drive 4, Singapore 117543, Singapore; b Department of Chemistry, Faculty of Science, National University of Singapore, 3 Science Drive 3, Singapore 117543, Singapore

## Abstract

In the title compound, C_7_H_7_N_5_, the non-H atoms are almost coplanar (r.m.s. deviation = 0.050 Å), with the N atom of pyridine ring oriented to the N—N(H) side of the 1,2,4-triazole ring. The mean planes of the pyridine and 1,2,4-triazole rings form a dihedral angle of 5.58 (7)°. The N atom of the amino group adopts a pyramidal configuration. The mol­ecules are linked into a two-dimensional network parallel to (10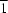) by N—H⋯N hydrogen bonds.

## Related literature

For 1,2,4-triazol-5-amines as building blocks in the synthesis of fused heterocyclic systems, see: Dolzhenko *et al.* (2006 ▶, 2007*a* ▶,*b* ▶); Fischer, (2007 ▶). For a summary of structural data for 1,2,4-triazoles, see: Buzykin *et al.* (2006 ▶). For crystal structures of Cu^II^ complexes with 3-pyridin-2-yl-1,2,4-triazol-5-amine, see: Ferrer *et al.* (2004 ▶).
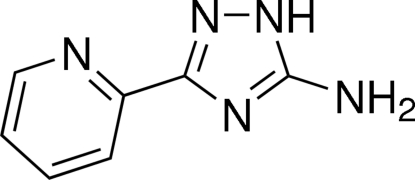


## Experimental

### 

#### Crystal data

C_7_H_7_N_5_
*M* *_r_* = 161.18
Monoclinic, 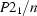

*a* = 7.3863 (6) Å
*b* = 7.9096 (6) Å
*c* = 13.2157 (11) Å
β = 91.832 (2)°
*V* = 771.70 (11) Å^3^
*Z* = 4
Mo *K*α radiation
μ = 0.10 mm^−1^
*T* = 223 (2) K
0.36 × 0.16 × 0.12 mm

#### Data collection

Bruker SMART APEX CCD diffractometer
Absorption correction: multi-scan (*SADABS*; Sheldrick, 2001 ▶) *T*_min_ = 0.967, *T*_max_ = 0.989
5336 measured reflections
1772 independent reflections
1519 reflections with *I* > 2σ(*I*)
*R*_int_ = 0.026

#### Refinement

*R*[*F* ^2^ > 2σ(*F* ^2^)] = 0.042
*wR*(*F* ^2^) = 0.110
*S* = 1.05
1772 reflections
121 parameters
H atoms treated by a mixture of independent and constrained refinement
Δρ_max_ = 0.21 e Å^−3^
Δρ_min_ = −0.20 e Å^−3^

### 

Data collection: *SMART* (Bruker, 2001 ▶); cell refinement: *SAINT* (Bruker, 2001 ▶); data reduction: *SAINT*; program(s) used to solve structure: *SHELXS97* (Sheldrick, 2008 ▶); program(s) used to refine structure: *SHELXL97* (Sheldrick, 2008 ▶); molecular graphics: *SHELXTL* (Sheldrick, 2008 ▶); software used to prepare material for publication: *SHELXTL*.

## Supplementary Material

Crystal structure: contains datablocks I, global. DOI: 10.1107/S1600536808042177/ci2719sup1.cif

Structure factors: contains datablocks I. DOI: 10.1107/S1600536808042177/ci2719Isup2.hkl

Additional supplementary materials:  crystallographic information; 3D view; checkCIF report

## Figures and Tables

**Table 1:** Hydrogen-bond geometry (Å, °)

*D*—H⋯*A*	*D*—H	H⋯*A*	*D*⋯*A*	*D*—H⋯*A*
N2—H2*N*⋯N5^i^	0.90 (2)	2.01 (2)	2.9010 (16)	171 (1)
N4—H4*A*⋯N3^ii^	0.90 (2)	2.11 (2)	2.9971 (16)	172 (1)
N4—H4*B*⋯N1^i^	0.93 (2)	2.19 (2)	3.0264 (16)	151 (1)

Symmetry codes: (i) 
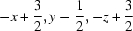
; (ii) 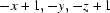.

**Fig. 1.**
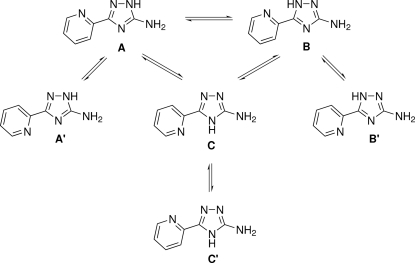
Possible tautomers and rotamers of 3-pyridin-2-yl-1,2,4-triazol-5-amine.

**Fig. 2.**
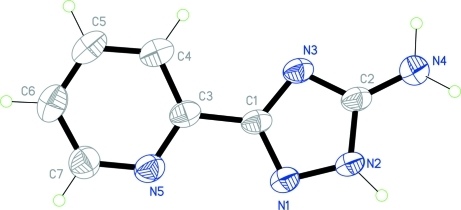
The molecular structure of the title compound, with the atomic numbering scheme. Displacement ellipsoids are drawn at the 50% probability level.

**Fig. 3.**
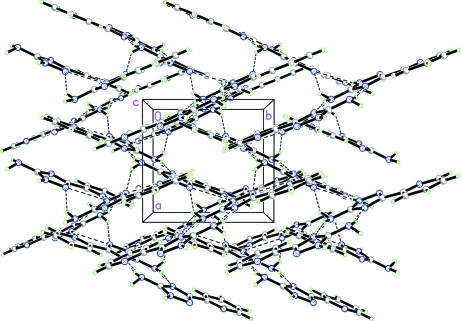
Molecular packing of the title compound, viewed along the c axis.

## supplementary crystallographic information

### Comment

1,2,4-Triazol-5-amines have been recognized as valuable synthons for the construction of fused heterocyclic systems, *e.g.* 1,2,4-triazolo[1,5-*a*]pyrimidines (Fischer, 2007) and 1,2,4-triazolo[1,5-*a*][1,3,5]triazines (Dolzhenko *et al.*, 2006). It also should be mentioned that 1,2,4-triazol-5-amines are widely used as ligands and crystallographic data on three different mononuclear complexes of 3-pyridin-2-yl-1,2,4-triazol-5-amine with Cu^II^ have been reported by Ferrer *et al.* (2004). However, no crystallographic study has been performed on the ligand.

In continuation of our investigations on using 1,2,4-triazol-5-amines in the synthesis of fused heterocyclic systems (Dolzhenko *et al.*, 2007*a*,b), we report herein the crystal structure of a synthetically important building block *viz.* 3-pyridin-2-yl-1,2,4-triazol-5-amine.

Due to annular tautomerism, 3-pyridin-2-yl-1,2,4-triazol-5-amine may theoretically exist in three tautomeric forms (A, B and C) and for each of them, rotameric structures A', B' and C' are possible (Fig.1). As observed in reported Cu^II^ complexes (Ferrer *et al.*, 2004), 3-pyridin-2-yl-1,2,4-triazol-5-amine was the only tautomeric form found in the crystal (Fig. 2). However, the molecule exists in the crystal as rotamer A in contrast to rotamer A' found in Cu^II^ complexes.

Bond lengths and angles in the molecule of 3-pyridin-2-yl-1,2,4-triazol-5-amine are within normal ranges, and comparable with values summarized for 1,2,4-triazoles by Buzykin *et al.* (2006). 3-Pyridin-2-yl-1,2,4-triazol-5-amine has practically planar geometry with slight deviation of the pyridyl moiety, which makes a dihedral angle of 5.58 (7)° with mean plane of the 1,2,4-triazole ring. The nitrogen atom (N4) of the amino group adopts a pyramidal configuration with 0.26 (2) Å deviation of the nitrogen atom from the C2/H4A/H4B plane.

The molecules are linked into a two-dimensional network parallel to the (101) by N—H···N hydrogen bonds (Table 1 and Fig.3).

### Experimental

3-Pyridin-2-yl-1,2,4-triazol-5-amine was prepared according to general method reported by Dolzhenko *et al.* (2007*a*,b). Single crystals suitable for crystallographic analysis were grown by recrystallization from ethanol.

### Refinement

N-bound H-atoms were located in a difference map and refined freely [N—H = 0.90 (2)–0.92 (2) Å]. C-bound H atoms were positioned geometrically (C—H = 0.94 Å) and were constrained in a riding motion approximation with *U*_iso_(H) = 1.2*U*_eq_(C).

### Crystal data

**Table:**

C_7_H_7_N_5_	*F*(000) = 336
*M**_r_* = 161.18	*D*_x_ = 1.387 Mg m^−^^3^
Monoclinic, *P*2_1_/*n*	Melting point: 493 K
Hall symbol: -P 2yn	Mo *K*α radiation, λ = 0.71073 Å
*a* = 7.3863 (6) Å	Cell parameters from 1841 reflections
*b* = 7.9096 (6) Å	θ = 3.0–26.6°
*c* = 13.2157 (11) Å	µ = 0.10 mm^−^^1^
β = 91.832 (2)°	*T* = 223 K
*V* = 771.70 (11) Å^3^	Block, colourless
*Z* = 4	0.36 × 0.16 × 0.12 mm

### Data collection

**Table:**

Bruker SMART APEX CCD diffractometer	1772 independent reflections
Radiation source: fine-focus sealed tube	1519 reflections with *I* > 2σ(*I*)
graphite	*R*_int_ = 0.026
φ and ω scans	θ_max_ = 27.5°, θ_min_ = 3.0°
Absorption correction: multi-scan (*SADABS*; Sheldrick, 2001)	*h* = −9→9
*T*_min_ = 0.967, *T*_max_ = 0.989	*k* = −8→10
5336 measured reflections	*l* = −14→17

### Refinement

**Table:**

Refinement on *F*^2^	Primary atom site location: structure-invariant direct methods
Least-squares matrix: full	Secondary atom site location: difference Fourier map
*R*[*F*^2^ > 2σ(*F*^2^)] = 0.042	Hydrogen site location: inferred from neighbouring sites
*wR*(*F*^2^) = 0.110	H atoms treated by a mixture of independent and constrained refinement
*S* = 1.05	*w* = 1/[σ^2^(*F*_o_^2^) + (0.0539*P*)^2^ + 0.1487*P*] where *P* = (*F*_o_^2^ + 2*F*_c_^2^)/3
1772 reflections	(Δ/σ)_max_ = 0.001
121 parameters	Δρ_max_ = 0.21 e Å^−^^3^
0 restraints	Δρ_min_ = −0.20 e Å^−^^3^

### Special details

**Table:**

Geometry. All e.s.d.'s (except the e.s.d. in the dihedral angle between two l.s. planes) are estimated using the full covariance matrix. The cell e.s.d.'s are taken into account individually in the estimation of e.s.d.'s in distances, angles and torsion angles; correlations between e.s.d.'s in cell parameters are only used when they are defined by crystal symmetry. An approximate (isotropic) treatment of cell e.s.d.'s is used for estimating e.s.d.'s involving l.s. planes.
Refinement. Refinement of *F*^2^ against ALL reflections. The weighted *R*-factor *wR* and goodness of fit *S* are based on *F*^2^, conventional *R*-factors *R* are based on *F*, with *F* set to zero for negative *F*^2^. The threshold expression of *F*^2^ > σ(*F*^2^) is used only for calculating *R*-factors(gt) *etc*. and is not relevant to the choice of reflections for refinement. *R*-factors based on *F*^2^ are statistically about twice as large as those based on *F*, and *R*- factors based on ALL data will be even larger.

### Fractional atomic coordinates and isotropic or equivalent isotropic displacement parameters (Å^2^)

**Table:**

	*x*	*y*	*z*	*U*_iso_*/*U*_eq_	
N1	0.72741 (16)	0.34999 (14)	0.68693 (8)	0.0383 (3)	
N2	0.66107 (16)	0.19639 (15)	0.71630 (8)	0.0393 (3)	
H2N	0.669 (2)	0.164 (2)	0.7817 (14)	0.052 (5)*	
N3	0.61324 (14)	0.20352 (14)	0.55193 (8)	0.0345 (3)	
N4	0.51489 (16)	−0.04181 (15)	0.64256 (9)	0.0398 (3)	
H4A	0.488 (2)	−0.095 (2)	0.5842 (13)	0.049 (4)*	
H4B	0.564 (2)	−0.106 (2)	0.6948 (13)	0.050 (4)*	
N5	0.80901 (15)	0.62905 (15)	0.56803 (8)	0.0388 (3)	
C1	0.69411 (16)	0.34759 (16)	0.58805 (9)	0.0331 (3)	
C2	0.59494 (16)	0.11150 (17)	0.63532 (9)	0.0343 (3)	
C3	0.74353 (16)	0.48955 (17)	0.52229 (9)	0.0343 (3)	
C4	0.72299 (19)	0.47748 (19)	0.41755 (10)	0.0425 (3)	
H4	0.6758	0.3786	0.3873	0.051*	
C5	0.77288 (19)	0.6128 (2)	0.35870 (11)	0.0491 (4)	
H5	0.7598	0.6076	0.2878	0.059*	
C6	0.8419 (2)	0.7554 (2)	0.40516 (12)	0.0486 (4)	
H6	0.8781	0.8489	0.3668	0.058*	
C7	0.85663 (19)	0.75796 (19)	0.50930 (12)	0.0455 (4)	
H7	0.9029	0.8561	0.5408	0.055*	

### Atomic displacement parameters (Å^2^)

**Table:**

	*U*^11^	*U*^22^	*U*^33^	*U*^12^	*U*^13^	*U*^23^
N1	0.0476 (6)	0.0387 (6)	0.0282 (5)	−0.0007 (5)	−0.0076 (4)	0.0006 (4)
N2	0.0518 (7)	0.0392 (6)	0.0261 (6)	−0.0013 (5)	−0.0092 (5)	0.0000 (5)
N3	0.0362 (5)	0.0396 (6)	0.0273 (5)	0.0042 (4)	−0.0059 (4)	−0.0028 (4)
N4	0.0498 (7)	0.0397 (6)	0.0291 (6)	−0.0021 (5)	−0.0110 (5)	−0.0001 (5)
N5	0.0420 (6)	0.0409 (6)	0.0332 (6)	0.0030 (5)	−0.0043 (5)	0.0012 (5)
C1	0.0330 (6)	0.0385 (7)	0.0273 (6)	0.0057 (5)	−0.0056 (5)	−0.0035 (5)
C2	0.0357 (6)	0.0391 (7)	0.0274 (6)	0.0053 (5)	−0.0068 (5)	−0.0030 (5)
C3	0.0305 (6)	0.0419 (7)	0.0301 (6)	0.0076 (5)	−0.0028 (5)	−0.0010 (5)
C4	0.0442 (7)	0.0522 (8)	0.0310 (7)	0.0046 (6)	−0.0016 (5)	−0.0025 (6)
C5	0.0488 (8)	0.0683 (10)	0.0304 (7)	0.0080 (7)	0.0032 (6)	0.0071 (7)
C6	0.0444 (8)	0.0558 (9)	0.0457 (8)	0.0057 (7)	0.0046 (6)	0.0150 (7)
C7	0.0454 (8)	0.0447 (8)	0.0460 (8)	0.0013 (6)	−0.0035 (6)	0.0050 (6)

### Geometric parameters (Å, °)

**Table:**

N1—C1	1.3221 (16)	N5—C3	1.3410 (17)
N1—N2	1.3708 (16)	C1—C3	1.4729 (18)
N2—C2	1.3422 (16)	C3—C4	1.3909 (18)
N2—H2N	0.902 (19)	C4—C5	1.380 (2)
N3—C2	1.3312 (17)	C4—H4	0.94
N3—C1	1.3656 (16)	C5—C6	1.374 (2)
N4—C2	1.3538 (18)	C5—H5	0.94
N4—H4A	0.896 (18)	C6—C7	1.377 (2)
N4—H4B	0.925 (17)	C6—H6	0.94
N5—C7	1.3354 (18)	C7—H7	0.94
			
C1—N1—N2	102.14 (10)	N5—C3—C4	122.10 (13)
C2—N2—N1	109.99 (11)	N5—C3—C1	116.99 (11)
C2—N2—H2N	129.2 (11)	C4—C3—C1	120.91 (12)
N1—N2—H2N	120.8 (11)	C5—C4—C3	119.01 (14)
C2—N3—C1	102.81 (10)	C5—C4—H4	120.5
C2—N4—H4A	116.4 (10)	C3—C4—H4	120.5
C2—N4—H4B	112.7 (10)	C6—C5—C4	119.12 (14)
H4A—N4—H4B	117.2 (14)	C6—C5—H5	120.4
C7—N5—C3	117.65 (12)	C4—C5—H5	120.4
N1—C1—N3	115.02 (12)	C5—C6—C7	118.36 (14)
N1—C1—C3	122.04 (12)	C5—C6—H6	120.8
N3—C1—C3	122.94 (11)	C7—C6—H6	120.8
N3—C2—N2	110.03 (12)	N5—C7—C6	123.76 (15)
N3—C2—N4	127.19 (11)	N5—C7—H7	118.1
N2—C2—N4	122.71 (12)	C6—C7—H7	118.1

### Hydrogen-bond geometry (Å, °)

**Table:**

*D*—H···*A*	*D*—H	H···*A*	*D*···*A*	*D*—H···*A*
N2—H2N···N5^i^	0.90 (2)	2.01 (2)	2.9010 (16)	171 (1)
N4—H4A···N3^ii^	0.90 (2)	2.11 (2)	2.9971 (16)	172 (1)
N4—H4B···N1^i^	0.93 (2)	2.19 (2)	3.0264 (16)	151 (1)

Symmetry codes: (i) −*x*+3/2, *y*−1/2, −*z*+3/2; (ii) −*x*+1, −*y*, −*z*+1.
